# Effectiveness of a multicomponent intervention to promote physical activity among Japanese remote workers: a pilot open-label randomized controlled trial

**DOI:** 10.1093/joccuh/uiae052

**Published:** 2024-09-05

**Authors:** Jihoon Kim, Ryoko Mizushima, Masahiro Morimoto, Yukako Fujita, Saki Shibuichi, Mafuyu Kato, Masahiko Gosho, Yoshio Nakata

**Affiliations:** Graduate School of Comprehensive Human Sciences, University of Tsukuba, 1-1-1 Tennodai, Tsukuba, Ibaraki 305-8574, Japan; Institute of Health and Sport Sciences, University of Tsukuba, Laboratory of Advanced Research D606, 1-1-1 Tennodai, Tsukuba, Ibaraki 305-8574, Japan; Institute of Health and Sport Sciences, University of Tsukuba, Laboratory of Advanced Research D606, 1-1-1 Tennodai, Tsukuba, Ibaraki 305-8574, Japan; Risk Management Department, 4th, MS &AD InterRisk Research & Consulting, Inc., Waterras Annex (10F & 11F), 2-105, Kanda Awajicho, Chiyoda-ku, Tokyo 101-0063, Japan; Wellbeing Management Department, PhoneAppli Inc., Hulic Kamiyacho (8F), 4-3-13, Toranomon, Minato-ku, Tokyo 105-0001, Japan; Human Capital Department, PhoneAppli Inc., Hulic Kamiyacho (8F), 4-3-13, Toranomon, Minato-ku, Tokyo 105-0001, Japan; Human Capital Department, PhoneAppli Inc., Hulic Kamiyacho (8F), 4-3-13, Toranomon, Minato-ku, Tokyo 105-0001, Japan; Department of Biostatistics, Institute of Medicine, University of Tsukuba, 1-1-1 Tennodai, Tsukuba 305-8575, Japan; Institute of Health and Sport Sciences, University of Tsukuba, Laboratory of Advanced Research D606, 1-1-1 Tennodai, Tsukuba, Ibaraki 305-8574, Japan

**Keywords:** occupational groups, health promotion, physical activity, randomized controlled trial

## Abstract

Objectives: Remote work (ie, teleworking) may adversely affect physical activity (PA) among workers, but no strategies have been effectively implemented to address this issue. We aimed to test whether a multicomponent intervention program could promote the PA of remote workers.

Methods: This study was an 8-week pilot open-label randomized controlled trial. Fifty-one participants (19 women) aged 23-58 years were recruited via an information technology company in Tokyo, Japan, and randomly assigned to the control (*n* = 26) or intervention (*n* = 25) group. The intervention group was provided a multicomponent intervention that comprised individual (lecture, print material, goal setting, feedback, and posters), sociocultural (supportive atmosphere and team building), and organizational (encouraging message from an executive) strategies. The control group only received posters. The primary outcome was an 8-week change in objectively measured moderate-to-vigorous PA (MVPA). The secondary outcomes were changes in light PA, moderate PA, vigorous PA, steps, and sedentary time. We also conducted subdomain analyses divided into working and nonworking days.

Results: No significant difference was observed in MVPA changes between the 2 groups. However, the intervention group showed significant improvement in light PA by +14.0 min/d (95% CI, 1.7-26.2). Subdomain analyses also showed no significant differences in MVPA changes between the 2 groups. However, MVPA in the intervention group significantly increased by +9.4 min/d (95% CI, 2.5-16.2) on working days.

Conclusions: The present multicomponent intervention was feasible for remote workers, although some revisions are necessary to enhance the effect size.

## Teaser

Key points: What is already known on this topic: Previous studies have reported that remote work might lead to a decrease in physical activity among workers, but there is a lack of research on adequate countermeasures in Japan.

What this study adds: This study suggests that a multicomponent intervention combining individual, sociocultural, and organizational strategies might promote physical activity-related outcomes among remote workers on working days.

How this study might affect research, practice, or policy: In Japan, where remote work is becoming more common, this study can help labor unions and the government develop countermeasures against the decrease in physical activity resulting from remote work.

## 1. Introduction

Coronavirus disease 2019 (COVID-19) is a respiratory syndrome with symptoms including fever, cough, and dyspnea.^1^ The World Health Organization declared the outbreak a global pandemic on March 11, 2020, after which the disease spread rapidly worldwide.^2^^,^^3^ The COVID-19 pandemic has led to lockdowns or social distancing, which have had a significant impact on individuals in terms of work, transportation, and leisure activities.^4^^,^^5^ A noticeable change was the spread of remote work (ie, “work from home” or teleworking) to minimize contact.^6^ Japan did not institute a lockdown but showed an increase (from 10% to 28%) in remote work similar to other countries.^7^ Moreover, a Tokyo Metropolitan Government report in March 2024 showed that 43.4% of companies in Tokyo were conducting remote work, and 45.6% of these companies conduct it 3 or more times a week.^8^ Therefore, it seems that various types of remote work, including hybrid remote work, have been established.

Previous studies have shown that remote work during the COVID-19 pandemic led to a decrease in physical activity (PA) and an increase in sedentary time (ST) among workers.^9^^-^^11^ Wilms et al^11^ conducted a systematic review and reported a reduction in moderate-to-vigorous PA (MVPA; range, −35% to 0%), light PA (LPA; range, −47% to −4%), and an increase in ST (range, −3% to 67%). Low PA and high ST have been identified as risk factors for various health conditions, including noncommunicable diseases and mental health issues.^12^ It is also reported that an acute decrease in PA may cause a decline in insulin sensitivity along with a loss of muscle mass and strength.^13^ Therefore, it is important to develop strategies that promote PA and reduce ST among remote workers to mitigate these risks.

Conventionally, workplace-based interventions have proven effective in improving PA and reducing ST.^14^ These interventions often comprise a multicomponent approach, considering the social-ecological model, and may include components such as exercise programs, sit-stand workstations, group support, stair-use promotion, and posters.^15^^-^^17^ However, due to the COVID-19 pandemic and the shift to remote work, it is uncertain whether these conventional intervention methods can be effectively implemented in a different work environment. Therefore, timely strategies that take into account the unique context of remote work are required to improve PA.

Previously, we developed a multicomponent PA intervention program using focus-group interviews, which was intended to understand office workers’ perceptions of PA and their needs.^18^ Then, the feasibility of a multicomponent PA intervention program was evaluated.^19^ The program was based on the social-ecological model and comprised individual (lectures, print materials, goal setting, and feedback), sociocultural (supportive atmosphere and team building), physical (posters), and organizational (encouraging messages from an executive) strategies. We provided the program remotely to office workers who commuted to their office and/or worked remotely in a hybrid manner. This 1-arm trial identified a difference of approximately 25 minutes of MVPA between commute workdays and remote workdays (59.8 min/d vs 35.1 min/d; *P* < .001). Additionally, participants significantly increased MVPA by 7.3 min/d during a week and increased MVPA by 7.1 min/d during remote workdays.^19^ Despite our previous study lacking a control group, these results led us to hypothesize that our intervention program could be implemented for fully remote workers. Therefore, this study aimed to examine the effectiveness and feasibility of our multicomponent intervention program in promoting PA among Japanese remote workers.

**Figure 1:**
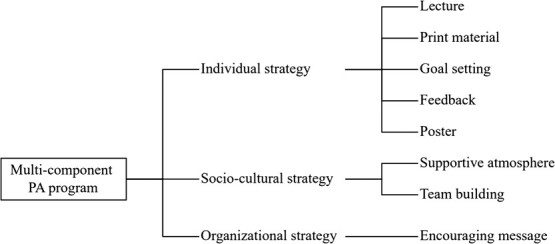
Multicomponent intervention program.

**Figure 2:**
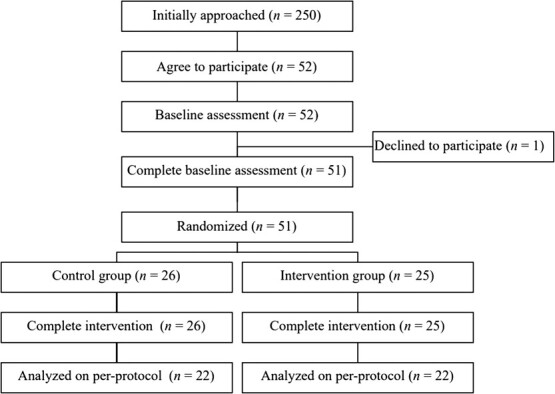
Flowchart of the present study.

## 2. Methods

### 2.1. Study design, participants, and randomization

This pilot open-label randomized controlled trial was conducted between January and March 2022 in areas around Tokyo, Japan. The study was approved by the University of Tsukuba Faculty of Health and Sport Sciences Ethics Committee (approval number Tai 021-188) and was registered in the University Hospital Medical Information Network Clinical Trials Registry (UMIN000046638) on January 15, 2022. The study adhered to the CONSORT 2010 statement and its extension to randomized pilot and feasibility trials.^20^^,^^21^

Participants were recruited from an information technology company in Tokyo, Japan, which engages in cloud services and application development. Most participants worked remotely; however, some commuted depending on their department or personal decisions. Eligible participants ranged between 20 and 65 years of age. Participants were excluded if they planned to retire permanently or temporarily during the study, could not use a smartphone, or had gait disturbances. Written informed consent was obtained from all the participants before the commencement of the study, and they were not compensated financially.

Following baseline assessments, participants were randomly assigned to either the control or the intervention group using a completely randomized design with a block size of 4. A biostatistician, M.G., generated a random number sequence, and the participating company's research staff allocated the participants. Concealment was performed until the participant allocation.

### 2.2. Intervention program

To promote PA, we provided the intervention group with an 8-week multicomponent program. Referring to our previous studies,^18^^,^^19^ we collaborated with the participating company to design the program, which included individual (lecture, print material, goal setting, feedback, and poster), sociocultural (supportive atmosphere and team building), and organizational (encouraging message from an executive) strategies (Figure 1). The intervention was delivered remotely, and the control group received the minimum intervention, only posters.

#### 2.2.1. Individual strategy

The participants received various components to promote PA, including lectures, print materials, goal setting, feedback, and posters. In Week 1, the participants received a 30-minute online motivational lecture and print materials from a researcher (Y.N.), who possesses vast experience in delivering lectures on PA and health, covering several topics such as the definition of PA, evidence supporting “+10 min of PA per day,” how to set appropriate exercise intensity, the difference between physical inactivity and ST, and the importance of breaking ST in daily routines.^22^^-^^24^ Additionally, participants received tips on setting goals to improve their PA. From Week 2 to Week 8, a researcher (Y.N.) provided weekly messages to motivate the participants and addressed any questions they had. The participants also received PA and ST summary reports that included information on their daily steps, activity score, and number of sit-to-stand transitions, which was measured using the ActivPAL device (PAL Technologies, Glasgow, UK). Finally, PA prompt posters were provided to the participants via the company's internal chat system. These posters were delivered only to participants working remotely and included information on the health risks associated with prolonged ST (Week 1), the health benefits of replacing sitting with standing (Week 3), the health benefits of walking (Week 5), and the importance of goal setting for improving PA (Week 7). Although providing PA posters was originally classified as a physical environment strategy in our previous study,^18^^,^^19^ we classified it as an individual strategy in the present study because they were delivered only via the internal chat system and not in the on-site workplace.

#### 2.2.2. Sociocultural strategy

The supportive atmosphere and team building were the sociocultural strategies provided in this study. The participating company's research staff notified the intervention group of the intervention program's contents and schedule. To promote a supportive atmosphere, they facilitated communication between participants and researchers, relaying participants' comments and questions regarding the weekly messages to a researcher (Y.N.). In turn, the researcher addressed participants' questions in the subsequent weekly messages. In Weeks 3 and 6, the participants engaged in both individual and team-based step-count competitions using smartphone applications provided by the company for team building. The research staff assigned approximately 4 participants per team. The results of the individual and team-based competitions were shared with participants via an internal chat room in Weeks 4 and 7, respectively.

**Table 1:** Characteristics of the participants at baseline.^a^

	**Intervention** **(*n* = 25)**	**Control** **(*n* = 26)**	**Total** **(*n* = 51)**
**Age, y**	38 (10)	38 (9)	38 (10)
**Women, *n* (%)**	9 (36)	10 (39)	19 (37)
**Height, cm**	169 (8)	167 (10)	168 (9)
**Weight,** ^**b**^ **kg**	69 (18)	64 (17)	67 (17)
**Body mass index,** ^**b**^ **kg/m**^**2**^	24 (5)	22 (4)	23 (4)
**Current smoker, *n* (%)**	7 (28)	4 (15)	11 (22)
**Four-year college graduate *n* (%)**	17 (68)	21 (81)	38 (75)
**Continuous years of service**	2 (0-9)	3 (0-10)	3 (0-10)
**Job position, *n* (%)**			
**Employee**	16 (64)	13 (50)	29 (57)
**Manager**	9 (36)	13 (50)	22 (43)
**Living with one or other people, *n* (%)**	15 (60)	20 (77)	35 (69)
**Currently married, *n* (%)**	11 (44)	15 (58)	26 (51)
**Remote working days (per 5-day working week)** ^**b**^			
**5**	18 (75)	13 (54)	31 (65)
**4**	3 (13)	8 (33)	11 (23)
**3**	0 (0)	1 (4)	1 (2)
**2**	1 (4)	0 (0)	1 (2)
**1**	1 (4)	1 (4)	2 (4)
**0**	1 (4)	1 (4)	2 (4)

a Data are presented as mean (SD) for age, height, and weight, median (range) for continuous years of service, and *n* (%) for categorical variables.

b *n* = 48 (intervention, *n* = 24; control, *n* = 24); missing data were mostly due to personal reasons (did not want to answer: intervention 1, control 2).

#### 2.2.3. Organizational strategy

An encouraging message from an executive was provided as an organizational strategy for the intervention group. This message encouraged active engagement in participation and was delivered in Week 1 through the internal chat system.

### 2.3. Measurements

#### 2.3.1. Baseline characteristics

Basic demographic information was collected from the participants using self-reported online questionnaires. We asked about age, sex, current smoking status (yes or no), educational attainment (4-year college graduate, college graduate, or high-school graduate or less), living arrangement (living alone or living with 1 or more other people), marital status (married or unmarried), and continuous years of service. The body mass index of each participant was calculated as the weight in kilograms divided by the square of their height in meters using their self-reported weight and height. Participants were given the option to skip any questions they did not wish to answer.

#### 2.3.2. Primary and secondary outcomes

The primary outcome of this study was a change in objectively measured MVPA from the baseline over the intervention period. The secondary outcomes were the changes in LPA, moderate PA (MPA), vigorous PA (VPA), number of steps, and ST. To measure these outcomes, participants were asked to wear a triaxial accelerometer (Active style Pro HJA-350IT; Omron Healthcare, Kyoto, Japan) on their waist for 7 consecutive days. The device uses a validated algorithm to count PA intensity (metabolic equivalents) and steps.^25^^,^^26^ For safety reasons, participants were instructed to remove the device during sleeping, bathing, swimming, or participating in contact sports. They were also requested to keep a daily diary about their waking-up time, bedtime, working time, break time, and type of work (remote or commuting). Participants were included in the analysis only if they had valid accelerometer data for at least 10 h/d and a minimum of 3 valid days.^27^

#### 2.3.3. Post-intervention survey

A post-intervention survey was conducted to evaluate the subjective satisfaction and effectiveness of the PA intervention program from the participants' perspective through an online assessment. The participants' satisfaction with the program was assessed using a 5-point satisfaction scale (very satisfied, satisfied, neither, unsatisfied, and very unsatisfied). The participants were also asked the following questions: (1) whether they knew about each component of the intervention or not; (2) whether they introduced each component of the intervention or not; and (3) whether they thought each component of the intervention was effective or not.

#### 2.3.4. Sample size and statistical analysis

Because this was a pilot trial, we did not carry out a formal sample size calculation. Data were analyzed according to the per-protocol principle, and any missing data were excluded. Data analysis was performed using SPSS version 28.0 (IBM Corp., Armonk, NY, USA), and a statistical significance level of 5% was set. Baseline characteristics of the intervention and control groups were compared using independent-sample *t* tests and χ^2^ analyses. Baseline characteristics were summarized as mean and SDs for continuous variables and *n* (%) for categorical variables. The primary and secondary outcomes were analyzed using the analysis of covariance (ANCOVA) model, with the treatment group as a factor and the relevant baseline value as a covariate.

Changes in PA outcomes within each group from baseline to Week 8 are shown as means and 95% CIs. Subdomain analyses were also conducted to assess the difference in effectiveness between working and nonworking days (eg, weekends or holidays).

## 3. Results

Figure 2 shows the flowchart of the participants. We initially contacted the information technology company with 250 remote workers and informed them regarding the recruitment of participants for our intervention program through an internal announcement. Among 250 workers in the participating company, 52 workers expressed interest in participating and provided written informed consent. During the baseline measurement, 1 participant declined the study due to illness. After baseline measurement, 51 participants were randomly allocated to either the control group (*n* = 26) or the intervention group (*n* = 25). All 51 participants completed the study. No adverse events were reported during the intervention period. Table 1 shows the baseline characteristics of the participants. The study population had a mean age of 38 ± 10 years and comprised 37% women. In total, 75% of the participants had a 4-year college degree, and the median duration of continuous service was 3 years (range, 0-10 years). Regarding baseline characteristics, no significant differences were observed between the control and intervention groups.

After excluding the invalid accelerometer data that were not assessed for at least 10 h/d and for a minimum of 3 valid days, 44 participants (control: *n* = 22; intervention: *n* = 22) were analyzed. No significant differences in baseline characteristics were found between the 44 participants who met the requirements (mean age 38 ± 10 years, 61% male, 18% smokers, and 55% married) and 7 who did not (mean age 38 ± 9 years, 71% male, 43% smokers, and 29% married).

Table 2 presents the changes in PA outcomes for 8 weeks. ANCOVA showed no significant between-group differences in MVPA between the intervention (+6.1 min/d; 95% CI, −0.8 to 12.9) and control (+3.0 min/d; 95% CI, −4.2 to 10.2) groups. However, the intervention group showed a significant improvement in LPA, with an increase of +14.0 min/d (95% CI, 1.7 to 26.2), but the difference between the 2 groups was not statistically significant. The intervention group showed improvement in MPA (+5.0 min/d), VPA (+1.1 min/d), steps (+459 steps/d), and ST (−17.1 min/d), but these improvements were not statistically significant. In the control group, MPA (+3.5 min/d) and steps (+615 steps/d) improved, but these improvements were not statistically significant.

**Table 2:** Changes in physical activity outcomes during 8 weeks for intervention and control groups.^a^

	**Intervention** **(*n* = 22)**			**Control** **(*n* = 22)**			**Between-groups *P* value** ^**b**^
**Outcomes**	**Baseline**	**Week 8**	**Change (95% CI)**	**Baseline**	**Week 8**	**Change (95% CI)**	
**MVPA, min/d**	32.9 (23.9)	39.0(29.9)	6.1(−0.8 to 12.9)	37.4(31.9)	40.3(26.8)	3.0(−4.2 to 10.2)	.607
**LPA, min/d**	159.3 (65.5)	173.2(68.1)	14.0(1.7 to 26.2)	166.4(59.6)	179.3(76.1)	12.9(−10.2 to 36.0)	.957
**MPA, min/d**	32.2 (23.4)	37.2(27.7)	5.0(−1.5 to 11.5)	34.6(29.1)	38.2(25.6)	3.5(−3.3 to 10.4)	.803
**VPA, min/d**	0.7 (1.3)	1.8(3.4)	1.1(−0.2 to2.3)	2.7(4.3)	2.2(3.6)	−0.6(−2.5 to 1.4)	.656
**Steps, steps/d**	3651 (2601)	4110(2553)	459(−246 to 1164)	4685(3762)	5299(3480)	615(−185 to 1414)	.472
**ST, min/d**	722.7 (132.8)	705.6 (149.9)	−17.1(−58.3 to 24.1)	638.9(82.1)	654.0(120.1)	15.1(−11.3 to 41.5)	.206
**Wearing time, min/d**	914.9 (113.0)	917.8 (129.3)	2.9(−33.6 to 39.5)	842.8(64.2)	873.7(107.7)	31.0(−4.1 to 66.0)	.360
**Valid days**	6.5 (0.9)	6.3(0.9)	−0.2(−0.6, 0.3)	6.2(1.1)	5.9(1.1)	−0.3(−0.8 to 0.3)	.302

Abbreviations: ANCOVA, analysis of covariance; LPA, light physical activity; MPA, moderate physical activity; MVPA, moderate-to-vigorous physical activity; ST, sedentary time; VPA, vigorous physical activity.

a Data are presented as mean (SD) for continuous variables.

b Between-group difference by ANCOVA including the treatment group as a factor and the relevant baseline value as a covariate.

Table 3 shows the result of subdomain analyses, which divided a week into working and nonworking days. Eight participants did not meet the requirements for wearing the accelerometer on nonworking days. A significant age difference was observed between the 36 participants who met the requirements (mean age 41 ± 10 years, 64% male, 14% smokers, and 61% married) and 8 who did not (mean age 31 ± 6 years, 50% male, 38% smokers, and 25% married) among baseline characteristics.

**Table 3:** Subdomain analyses in terms of working and nonworking days.^a^

	**Intervention**			**Control**			**Between-groups *P* value** ^**b**^
**Outcomes**	**Baseline**	**Week 8**	**Change (95% CI)**	**Baseline**	**Week 8**	**Change (95% CI)**	
***Working days*** ^***c***^							
**MVPA, min/d**	25.4(23.9)	34.7(27.3)	9.4(2.5 to 16.2)	29.1(28.4)	34.5(24.2)	5.5(−1.1 to 12.0)	.472
**LPA, min/d**	139.5(65.7)	152.4 (62.1)	12.9(−1.2 to 27.0)	141.6(58.2)	156.1 (64.2)	14.5(−2.0 to 31.0)	.853
**MPA, min/d**	24.9(23.3)	33.0(25.9)	8.1(1.6 to 14.6)	26.3(26.5)	32.4(22.8)	6.2(−1.2 to 13.5)	.714
**VPA, min/d**	0.4(1.1)	1.7(3.5)	1.2(−0.3 to 2.8)	2.8(4.8)	2.1(3.8)	−0.7(−2.9 to 1.5)	.816
**Steps, steps/d**	2700(2615)	3684 (2167)	984(195 to 1774)	3564(3272)	4459 (3057)	895(229 to 1561)	.774
**ST, min/d**	774.0 (129.7)	742.8 (154.5)	−31.2(−77.3 to 15.0)	691.6(93.1)	700.1 (121.5)	8.5(−26.3 to 43.4)	.270
**Wearing time, min/d**	938.9(112.6)	929.9(134.3)	−8.9(−55.4 to 37.5)	862.2(84.4)	890.7(106.5)	28.5(−10.6 to 67.6)	.526
**Valid days**	4.9(0.5)	4.6(0.7)	−0.2(−0.6 to 0.1)	4.7(0.6)	4.7(0.6)	0(−0.4 to 0.4)	.782
***Nonworking days*** ^***c***^							
**MVPA, min/d**	54.3(38.2)	52.3(45.1)	−1.9(−19.2 to 15.4)	61.8(49.1)	63.8(52.3)	1.9(−23.2 to 27.1)	.632
**LPA, min/d**	216.0(106.4)	230.2 (120.8)	14.3(−32.6 to 61.1)	244.9(75.0)	278.8 (121.4)	33.9(−31.6 to 99.3)	.412
**MPA, min/d**	52.8(36.1)	50.2(42.4)	−2.6(−18.7 to 13.6)	58.3(44.3)	61.4(50.2)	3.1(−19.9 to 26.2)	.559
**VPA, min/d**	1.5(4.0)	2.2(5.1)	0.6(−2.2 to 3.5)	3.6(8.4)	2.4(3.8)	−1.2(−5.8 to 3.4)	.980
**Steps, steps/d**	6294(4140)	5436 (4485)	−859(−2558 to 841)	7790(5813)	8800 (6549)	1010(−1802 to 3823)	.122
**ST, min/d**	597.4 (198.8)	610.8 (197.6)	13.3(−49.3 to 76.0)	503.3(93.7)	495.7 (161.7)	−7.6(−88.2 to 73.0)	.370
**Wearing time, min/d**	867.6(152.3)	893.3(181.4)	25.7(−27.0 to 78.4)	810.1(79.5)	838.3(132.3)	28.2(−45.1 to 101.5)	.876
**Valid days**	1.7(0.5)	1.8 (0.6)	0.1(−0.2 to 0.3)	1.7(0.6)	1.7(0.5)	0(−0.4 to 0.4)	.731

Abbreviations: ANCOVA, analysis of covariance; LPA, light physical activity; MPA, moderate physical activity; MVPA, moderate-to-vigorous physical activity; ST, sedentary time; VPA, vigorous physical activity.

a Data are presented as mean (SD) for continuous variables.

b Between-group difference by ANCOVA including the treatment group as a factor and the relevant baseline value as a covariate.

c Working days, *n* = 44 (intervention, *n* = 22; control, *n* = 22); nonworking days, *n* = 36 (intervention, *n* = 20; control, *n* = 16).

No significant differences between the intervention and control groups were revealed in all PA outcomes. However, significant increases were found in MVPA (+9.4 min/d; 95% CI, 2.5-16.2), MPA (+8.1 min/d; 95% CI, 1.6-14.6), and steps (+984 steps/d; 95% CI, 195-1774) in the intervention group on working days. On the contrary, only steps (+895 steps/d; 95% CI, 229-1516) increased in the control group on a working day. Meanwhile, no significant changes were found on nonworking days.

Table 4 presents the results of the post-intervention survey completed by the 51 participants who underwent the intervention. In the intervention group, 10, 9, and 6 participants responded “very satisfied,” “satisfied,” and “neither,” respectively. In contrast, in the control group, 8, 9, 8, and 1 participants responded “very satisfied,” “satisfied,” “neither,” and “very unsatisfied,” respectively. From the proportion of participants who answered “yes” for Q1-Q3, participants in the intervention group generally showed higher positive responses across multicomponent interventions compared with the control group.

**Table 4:** Post-intervention survey (*n* = 51).^a^

	**Q1. Were you aware of each intervention component during the intervention period?**		**Q2. Did you introduce each component of the intervention during the intervention period?**		**Q3. Do you think each intervention component was effective in improving physical activity?**	
**Intervention** **component**	**Intervention** **(*n* = 25)**	**Control** **(*n* = 26)**	**Intervention** **(*n* = 25)**	**Control** **(*n* = 26)**	**Intervention** **(*n* = 25)**	**Control** **(*n* = 26)**
**Lecture**	23 (92.0)	6 (23.1)	22 (88.0)	5 (19.2)	17 (68.0)	5 (19.2)
**Print material**	19 (69.2)	8 (30.8)	16 (64.0)	4 (15.4)	16 (64.0)	5 (19.2)
**Goal setting**	15 (60.0)	4 (15.4)	14 (56.0)	4 (15.4)	12 (48.0)	4 (15.4)
**Feedback**	16 (64.0)	4 (15.4)	13 (52.0)	4 (15.4)	12 (48.0)	4 (15.4)
**Poster**	21 (84.0)	13 (50.0)	11 (44.0)	3 (11.5)	13 (52.0)	3 (11.5)
**Supportive atmosphere**	24 (96.0)	19 (73.1)	18 (72.0)	12 (46.2)	19 (76.0)	14 (53.8)
**Team building**	9 (36.0)	9 (34.6)	7 (28.0)	4 (15.4)	7 (28.8)	3 (11.5)
**Encouraging message**	16 (64.0)	12 (46.2)	14 (56.0)	8 (30.8)	13 (52.0)	7 (26.9)

a Data answered “yes” are presented as *n* (%).

## 4. Discussion

This pilot randomized controlled trial aimed to assess the effectiveness of a multicomponent intervention program comprising individual (such as lecture, print material, goal setting, feedback, and posters), sociocultural environment (supportive atmosphere and team building), and organizational (an encouraging message from an executive) strategies in promoting PA among Japanese remote workers. The results showed no significant between-group differences in terms of changes in MVPA, which was the primary outcome, as well as other PA outcomes. However, significant improvement in LPA was observed in the intervention group, with a difference of +14.0 min/d. Notably, previous studies have shown that improvement in LPA has beneficial effects on obesity, blood lipid and glucose metabolism, and mortality.^28^

The improvement in LPA was consistent with the previous studies^29^^,^^30^ but was inconsistent with the results of our feasibility trial, wherein we observed a significant increase in MVPA.^19^ However, the present intervention group showed an increase in MVPA, MPA, and VPA, along with a decrease in ST, albeit not statistically significant. The control group also showed an increase in MVPA, LPA, MPA, and steps, but they demonstrated an increase in ST and a decrease in VPA.

The subdomain analysis, which divided the week into working and nonworking days, also did not show any significant between-group differences in PA outcomes between the intervention and control groups. On working days, the intervention group showed significant improvements in MVPA (9.4 min/d), MPA (8.1 min/d), and steps (984 steps/d). Improvements in LPA, VPA, and ST were also observed, although they were not statistically significant. In contrast, the control group showed a significant increase in steps (895 steps/d) and improvement in MVPA, LPA, and MPA but worsening of ST and VPA. A change of +9.4 min/d in MVPA (close to 10 minutes) on working days in the intervention group was a highly significant finding. An MVPA of +10 minutes has been reported to offer numerous benefits in reducing the risk of noncommunicable diseases, improving mental health, reducing the risk of musculoskeletal disease, and decreasing mortality.^22^^,^^31^ On nonworking days, the intervention group generally presented lower PA outcomes after the intervention compared with before, whereas the control group showed an increase in PA outcomes. These findings highlight the importance of considering the context and timing of PA intervention. Future interventions may need to consider the differences in PA patterns on working and nonworking days and tailor their strategies accordingly.

The results of the post-intervention survey explain why no between-group differences were observed in the present study. As previously mentioned, the intervention group received a multicomponent intervention program, whereas the control group only received minimal intervention via posters. However, the post-intervention survey revealed that the knowledge of participants in the control group regarding the multicomponent intervention program extended beyond the posters. Particularly, 73% of the control group participants were aware of the supportive atmosphere, 35% the team building, and 46% the encouraging messages. Furthermore, 46% and 31% of the control group participants reported implementing a supportive atmosphere and encouraging messages, respectively. From the post-intervention survey, we concluded that contamination, which is treatment diffusion (ie, contact with those in the treatment group and learning about the intervention or if the intervention is widely available outside the study),^32^ occurred in this study. Considering contamination in the future, a cluster randomized controlled trial should be necessary to draw more robust evidence.

The present study had some strengths. This study proved the feasibility of a non–face-to-face PA intervention program for Japanese remote workers during the COVID-19 restrictions. To our knowledge, only a few intervention studies have been conducted despite the widespread adoption of remote work.^7^^,^^8^ Moreover, very few studies have demonstrated decreased PA due to the adoption of remote work worldwide and in Japan.^9^^-^^11^ Although there were no significant between-group differences, improvement in MVPA (+9.4 min/d) during workdays in the intervention group suggests the feasibility of this study. The intervention program used in this study might be cost-effective compared with previous studies that involved providing incentives, sit-stand workstations, treadmill workstations, and wearable devices.^15^^-^^17^ We think our multicomponent intervention program is more feasible and likely to be accepted by most companies due to the low financial and occupational burden. This advantage is significant given the current economic and public health challenges being faced by many companies employing remote workers.

This study also had some limitations. Firstly, we found treatment diffusion in the present study. We conducted this study assuming that remote workers would not be influenced by the sociocultural and organizational environment. However, contrary to our expectations, participants in the control group were also affected by environmental interventions. A cluster randomized controlled trial is necessary to show the effectiveness of our multicomponent intervention program. Secondly, baseline characteristics indicated that the participants did not work remotely all the time (intervention, 75%; control, 54%). Additionally, we did not have any information on the 198 individuals who did not express willingness to participate. Therefore, we cannot show the differences between the 52 participants who expressed willingness and the remaining individuals. Therefore, the generalizability of this study is limited. Thirdly, this study has an issue with valid days for the subdomain analysis. A previous study recommended at least 3 valid days (2 weekdays and 1 weekend day) for measuring PA.^33^ However, the present study used data from at least 3 days without considering weekend data. Therefore, 8 participants were excluded because there were no data on nonworking days. Additionally, if they had data on nonworking days, the number of valid days mostly met the requirements of 1 or 2 days. The limited number of valid days may not be enough for representativeness. Furthermore, considering that 8 excluded participants were younger than those included in the subdomain analysis, selection bias exists, and caution is necessary when interpreting the results. Fourthly, we did not conduct mid-term measurements because of the relatively short intervention period and the burden on participants. We believe it is important to conduct mid-term measurements in future studies, considering the duration of the intervention. Fifthly, we did not perform a formal sample size calculation or adjust for multiplicity of testing because this was a pilot randomized trial. Finally, we provided an intervention program for remote workers, which was developed on the basis of our focus-group interviews^18^ and 1-arm intervention study^19^ conducted on office workers. In the future, it is necessary to conduct interviews or surveys with remote workers to create an intervention program that reflects the social-ecological situation of remote workers.

## 5. Conclusions

This study showed that the multicomponent PA intervention was feasible among Japanese remote workers, but there is a need to revise the program to enhance its effectiveness. The study also suggests that there is a potential for contamination in delivering the intervention program, which means that participants in the control group may have been exposed to some components of the intervention. Further long-term studies and cluster randomized control trials are needed to strengthen the evidence.

## Data Availability

Data from this study may be obtained from the authors upon reasonable request.
